# Safety and efficacy of the pan-FGFR inhibitor erdafitinib in advanced urothelial carcinoma and other solid tumors: A systematic review and meta-analysis

**DOI:** 10.3389/fonc.2022.907377

**Published:** 2023-01-26

**Authors:** Xinyi Zheng, Hang Wang, Junyue Deng, Minghe Yao, Xiuhe Zou, Fan Zhang, Xuelei Ma

**Affiliations:** ^1^ West China School of Medicine, Sichuan University, Chengdu, Sichuan, China; ^2^ Department of Biotherapy, West China Hospital, Sichuan University, Chengdu, Sichuan, China; ^3^ Department of Thyroid Surgery, West China Hospital, Sichuan University, Chengdu, Sichuan, China; ^4^ Health Management Center, General Practice Center, West China Hospital, Sichuan University, Chengdu, China

**Keywords:** erdafitinib, urothelial carcinoma, FGFR, hyperphosphatemia, central serous chorioretinopathy

## Abstract

**Objective:**

This review aimed to comprehensively analyze the safety and efficacy of erdafitinib in treating advanced and metastatic urothelial carcinoma and other solid tumors.

**Methods:**

PubMed, Embase, and ClinicalTrials.gov were searched until 10 February 2022. The safety outcome as adverse events and efficacy outcomes, including objective response rate, stable disease rates, and progressive disease rates, were selected and analyzed by comprehensive meta-analysis version 3.0 and STATA 15.0.

**Results:**

The most common all-grade adverse events were hyperphosphatemia, dry mouth, stomatitis, diarrhea, and dysgeusia. The occurrence of ≥3 adverse events was relatively low, and stomatitis and hyponatremia were the most common. Moreover, eye disorders could not be ignored. Efficacy in urothelial carcinoma patients was obviously better than in other solid tumor patients, with a higher objective response rate (0.38 versus 0.10) and lower progressive disease rate (0.26 versus 0.68). All responses occurred in patients with fibroblast growth factor receptor (FGFR) alteration. In those patients, a specific FGFR alteration (*FGFR3-TACC3*) was observed to have a maximum response.

**Conclusion:**

Erdafitinib has satisfactory clinical activity for metastatic urothelial carcinoma and other solid tumors, while the toxicity is acceptable. With more RCTs and combination therapy trials published, erdafitinib will be applied widely.

## 1 Introduction

Urothelial carcinoma (UC) refers to a transitional urothelial tumor of the urinary tract. It can be divided into upper and lower urothelial carcinoma according to the diseased region. Bladder cancer is the most common type of lower urothelial carcinoma, accounting for 90% of the total. Other types of urothelial carcinoma, such as renal pelvis and urethral carcinoma, are scarce. Generally, UC is the 10th most commonly diagnosed cancer worldwide, with approximately 573,000 new cases and 213,000 deaths in 2020 (1, 2). It has a predominance of male patients, with respective incidence and mortality rates of 9.5 and 3.3 per 100,000 among men, which are approximately four times those among women globally. For non-muscle-invasive bladder cancer, the present treatment is transurethral resection of bladder tumor (TURBT), intravesical chemotherapy, and intravesical BCG immunotherapy (3, 4). For unresectable or metastatic bladder tumor, platinum-based combination chemotherapies are the major therapy (5, 6). However, the efficacy of platinum-based drugs is not satisfactory, with a median survival of only 7.4 months. Since 2019, the application of FGFR inhibitors has innovated treatment options for advanced and metastatic UC, increasing the median survival by 3 months (7).

Erdafitinib is an ATP-competitive inhibitor of FGFR1–4. It is a small molecule inhibitor (SMi) that reversibly inhibits FGFR kinase autophosphorylation and decreases resultant downstream signaling (8). Under physiological conditions, fibroblast growth factor receptor 1–4 (FGFR1–4) bind to fibroblast growth factors (FGFs) to exert tyrosine kinase regulatory effects (9), which play a vital role in angiogenesis and damage repair. The FGFR molecule includes three extracellular immunoglobulin domains, one transmembrane domain, and one intracellular domain. The intracellular domain can activate the RAS-MAPK-ERK and PI3K-AKT pathways (10–14). However, gene amplification, mutation, rearrangement, or translocations occur and alter the signaling pathway, which leads to cell proliferation or migration (15–19).

The mechanism of erdafitinib inhibits these pathways from upstream, which can impede the growth of tumors. In a study, BLC2001(NCT02365597), those patients who had not responded to PD-1 treatment achieved an objective response with erdafitinib treatment. In other clinical trials (NCT01703481 and NCT01962532), erdafitinib resulted in prolonged progression-free survival and median duration of response. Therefore, the FDA granted approval to erdafitinib for metastatic urothelial carcinoma in patients with susceptible alterations in FGFR2 or FGFR3 who have progressed platinum-containing chemotherapy, including within neoadjuvant or adjuvant platinum-containing chemotherapy. It was also the first FGFR kinase inhibitor approved by FDA for urothelial carcinoma. However, erdafitinib causes adverse effects, such as increased serum phosphate, stomatitis, and central serous chorioretinopathy. These adverse events (AEs) may reduce medication compliance, which leads to reduced efficacy.

To our knowledge, there have not been any meta-analyses about the safety and efficacy of erdafitinib. To offer evidence-based references for physicians, we conducted this study to determine the most meaningful AEs and efficacy outcomes of erdafitinib.

## 2 Methods

### 2.1 Literature search

PRISMA (Preferred Reporting Items for Systematic reviews and Meta-Analyses) guidelines were followed to complete this meta-analysis. PubMed, Embase, ClinicalTrials.gov, Cochrane Library, and China National Knowledge Infrastructure (CNKI) were searched for clinical trials and related articles until 10 February 2022. In addition, the references of reviews or trials related to erdafitinib were screened to avoid the omission of valuable articles. There was no restriction to language. The following words were used for searching: “erdafitinib” or “JNJ-42756493” or “Balversa.”

### 2.2 Study selection

Two reviewers independently selected the search results according to PRISMA flow diagrams. Discrepancies were resolved by the third author. The inclusion criteria were as follows: (1) patients were confirmed to have carcinoma by pathology; (2) the gene alteration of patients was included in fusion, mutation, and amplification; (3) the interventions of studies included erdafitinib singly or combined with other drugs; and (4) relevant data of efficacy and safety were reported. Unrelated articles, case reports, retrospective studies, reviews, and studies that lacked necessary data or full text were excluded.

### 2.3 Data extraction

Data from the included articles were extracted independently by two reviewers while discussing disagreements with the third reviewers. Basic information, such as the first author’s name, publication year, clinical trial sequence number, study phase, study design, sample size, median age, median follow-up, carcinoma histology, and treatment regimens, was extracted. The efficacy indicators included the complete response rate (CRR), partial response rate (PRR), objective response rate (ORR, which referred to the presence of at least a confirmed complete response or confirmed partial response), stable disease (SD) rate, progressive disease rate (defined as >20% increase in the longest diameters of target lesions or the appearance of a new lesion), hazard ratio (HR), and risk ratio (RR). The data used for safety analyses were collected from all-grade and grade ≥3 AEs.

### 2.4 Statistical analysis

STATA was used to count the standard error of CR, PR, and ORR. We conducted a single-rate meta-analysis to draw a forest plot. Meanwhile, the odds ratio was calculated to compare erdafitinib with other treatments. Comprehensive Meta-Analysis (CMA) Version 3.0 was used to analyze all-grade and grade ≥3 AEs to calculate the event rate and 95% CI. STATA and CMA were used to analyze heterogeneity. *I* ^2^ > 50% and *p* < 0.05 were considered as high heterogeneity. A fixed-effects model was used for *I* ^2^ <50%; random-effects model analysis was used for *I* ^2^ > 50%.

If we included 10 more studies, STATA 15.0 was used to analyze the heterogeneity of the included literature. If there was high heterogeneity, the METAREG command was used for meta-regression analysis. We discussed the sources of heterogeneity. At the same time, if 10 more articles were included, Begg’s and Egger’s funnel plots were conducted to investigate publication bias.

### 2.5 Risk of bias and study quality

The risk of bias of randomized controlled trials was obtained by RevMan 5.4 (The Cochrane Collaboration). The articles were evaluated in the following processes: sequence generation, allocation concealment, blinding of participants and personnel, blinding of outcome assessment, incomplete outcome data, selective reporting, and others. For the nonrandomized studies, the bias was assessed by the risk of bias in nonrandomized studies of interventions (ROBINS-I) tool (20, 21). ROBINS-I included seven domains: allocation bias, selection bias, observer bias, performance bias, attrition bias, detection bias, and analysis reporting bias. Meanwhile, ROBINS-I was used to assess the quality of non-randomized studies.

## 3 Results

### 3.1 Study selection

**Figure 1:**
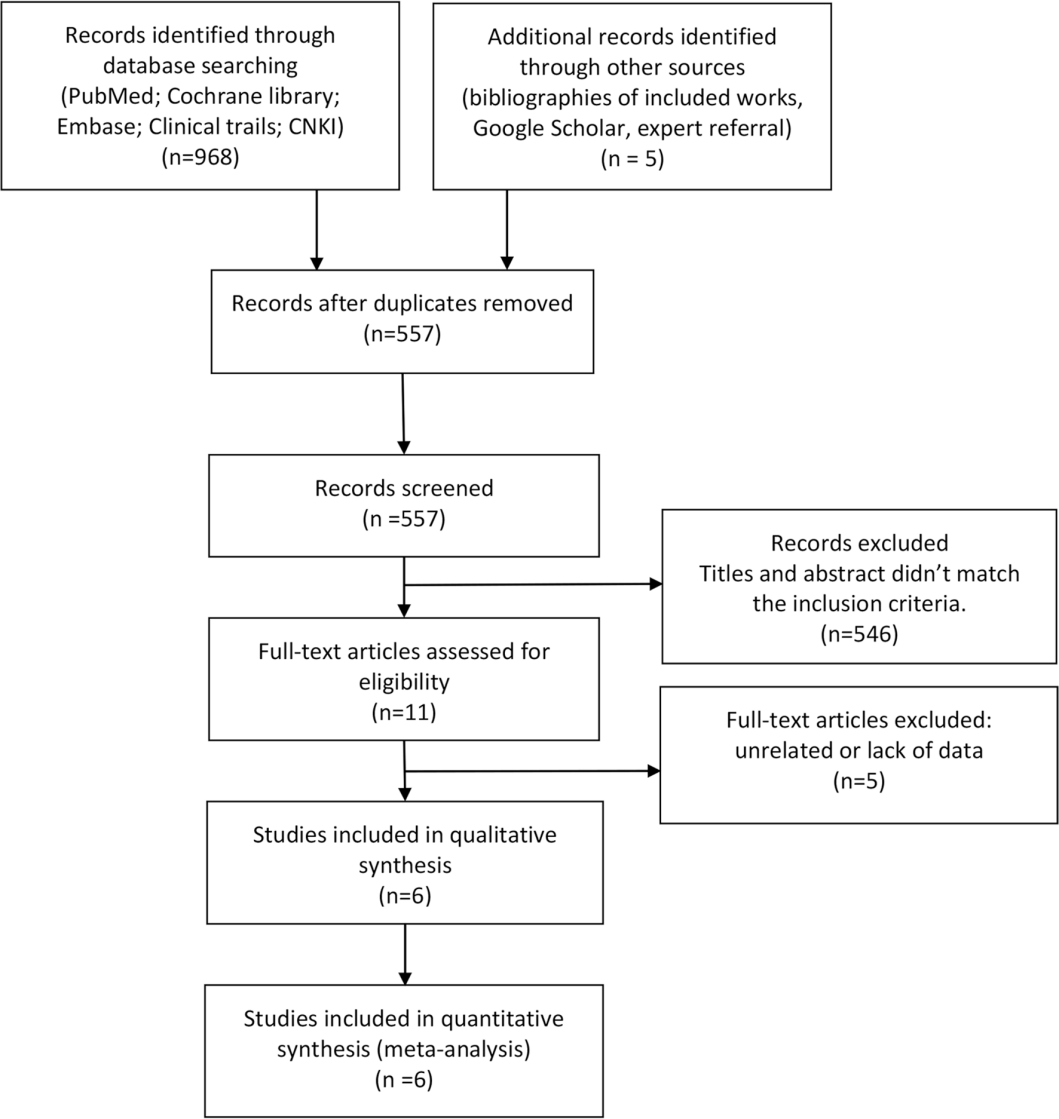
Flow diagram of literature selection for systemic reviews and meta-analyses (PRISMA).

A total of 968 articles were produced through the search strategy. Five articles were searched through the references of previous reviews. After removing duplications, 557 were screened based on the title and abstract, and 546 unrelated articles were excluded. Eleven studies were selected, but five articles lacked the necessary data. Finally, six trials were included. The study selection procedure is shown in **Figure 1**.

### 3.2 Characteristics of studies

The included studies were published from 2015 to 2022. There were three phase I and three phase II clinical trials, and all were nonrandomized. In phase I trials, patients with various kinds of solid tumors were included, in which some patients with UC were not being classified between other solid tumors. Meanwhile, every patient in phase II was suffering from urothelial carcinoma. Most of the included trials described FGFR alterations in patients except NCT01703481 (Tabernero,2015) and NCT01962532 (Nishina,2017). Mutations and fusions were the major gene alterations of FGFR. In NCT02365597 (Loriot,2019), the proportion of FGFR3 mutations was 74/99, while that of FGFR3 fusions was 25/99. In Bahleda’s research, the scale of FGFR mutations or fusions [mutation (+)/fusion (−) OR mutation (−)/fusion (+)] was 58/187, that of the amplifications was 45/187, and the ratio of co-alteration [mutation (+)/fusion (+)] was 5/187. In addition, all 12 patients in Monterio’s trial were found to have FGFR3 alterations. In NCT02365597 (Siefker-Radtke,2022), 70/101 were FGFR mutation (+)/fusion (−), 25/101 were mutation (−)/fusion (+), 6/101 were FGFR mutation (+)/fusion (+), and 5/101 were FGFR mutation/fusion co-alterations.

Erdafitinib was singly used for all six articles with constant, escalation, or intermittent doses. Most of the persistent doses ranged from 6 to 9 mg, while the intermittent dose was 10 or 12 mg. Notably, we found that in the latest trials, a constant dose of 8–9 mg was used more frequently, which might be related to the recommendation by the FDA. All the included articles used RECIST 1.1 (Response Evaluation Criteria in Solid Tumors) to assess the efficacy, while CTCAE 4.0–5.0 (Common Terminology Criteria for Adverse Events) was used for safety assessment. More basic information is displayed in **Table 1**.

**Table 1:** Basic characteristics of the included trials.

Author/Year	Clinical trials information	Study design	Study phase	Sample size	Median age (years)	Treatment	Treatment regimens	Median follow-up	Histology
Josep Tabernero, 2015	NCT01703481	Open-label, multicenter (escalating multiple dose cohorts)	I	65	59 (27–75)	Erdafitinib	0.5/2/4/6/9 mg qd==21 days OR 10/12 mg 7 days on+7 days off==28 days	8–16 weeks	Solid tumor (advance)
Tomohiro Nishina 2017	NCT01962532	Open-label, multicenter, single-arm, dose escalation	I	19	62.1	Erdafitinib	2/4/6 mg qd OR 10/12 mg qd [7 days on/off]	12 weeks	Advanced or refractory solid tumors
Rastislav Bahleda 2019	NCT01703481	Multicenter, escalating multiple-dose cohorts	I	187	60 (21–84)	Erdafitinib	9 mg qd==21days OR 10/12 mg qd (7 days on +7 days off==28 days)	24 weeks	Advanced or refractory solid tumors
Y. Loriot 2019	NCT02365597	Open-label	II	99	68 (36–87)	Erdafitinib	8–9 mg qd	24 months (IQR = 0.7–17.4)	Locally advanced and unresectable or metastatic urothelial carcinoma
Fernando Sabino M. Monteiro 2021	NA	Single-arm trial	II	12	76	Erdafitinib	8 mg qd	16.2 months	Metastatic urothelial carcinoma (mUC)
Arlene O Siefker-Radtke 2022	NCT02365597	Open-label, non-comparator	II	101	67 (61–73)	Erdafitinib	8–9 mg qd	24 months (IQR = 22.7–26.6)	Locally advanced and unresectable or metastatic urothelial carcinoma

### 3.3 Safety of erdafitinib

The rates of all-grade and grade ≥3 AEs were pooled from single-arm studies. The all-grade AEs are shown in **Table 2** and **Supplementary Material 1**. Among them, the top five most frequent AEs were dry mouth (42.4%, 95% CI 38.0%–46.9%), dysgeusia (30.8%, 95% CI 26.3%–35.7%), dry skin (30.6%, 95% CI 26.5%–34.9%), abnormal hepatic function (21.5%, 95% CI 13.2%–33.2%), and nausea (20.5%, 95% CI 17.1%–24.5%) in the fixed-effects model. In the random-effects model, hyperphosphatemia ranked first in incidence (68.2%, 95% CI 59.4%–75.8%), followed by stomatitis (37.4%, 95% CI 27.0%–49.0%), diarrhea (30.9%, 95% CI 18.8%–46.4%), decreased appetite (30.6%, 95% CI 22.3%–40.4%), and asthenia (27.7%, 95% CI 15.4%–44.7%). Eye disorders that might be caused by central serous chorioretinopathy cannot be ignored.

**Table 2:** The all-grade adverse events classified by CACTE 5.0 and the details.

Adverse events	No. of studies	No. of AE	No. of patients	Event rate with 95% CI	*p*-value	Model
Blood and lymphatic system disorders						
Anemia	4	56	284	0.199[0.156, 0.250]	0.000	Fixed
Eye disorders						
Blurred vision	5	54	418	0.137[0.106, 0.175]	0.000	Fixed
Cataract	1	6	99	0.061[0.027, 0.128]	0.000	Fixed
Dry eye	4	103	452	0.220[0.165, 0.286]	0.000	Random
Keratitis	1	5	99	0.051[0.021, 0.116]	0.000	Fixed
Blepharitis (eye disorders, other)	1	1	19	0.053[0.007, 0.294]	0.005	Fixed
Gastrointestinal disorders						
Abdominal pain	3	18	149	0.125[0.069, 0.218]	0.000	Fixed
Abdominal pain upper (abdominal pain)	1	8		0.123[0.063, 0.227]	0.000	Fixed
Angular cheilitis (cheilitis)	1	1	19	0.053[0.007, 0.294]	0.005	Fixed
Colitis	1	5		0.051[0.021, 0.116]	0.000	Fixed
Constipation	5	105	471	0.231[0.152, 0.335]	0.000	Random
Diarrhea #	6	160	483	0.309[0.188, 0.464]	0.017	Random
Dry mouth *	6	203	483	0.424[0.380, 0.469]	0.001	Fixed
Gastritis	1	1	19	0.053[0.007, 0.294]	0.005	Fixed
Gingivitis (periodontal disease)	1	1	19	0.053[0.007, 0.294]	0.005	Fixed
Stomatitis (mucositis oral) #	6	195	483	0.374[0.270, 0.490]	0.034	Random
Aphthous ulcer (mucositis oral)	1	4	99	0.040[0.015, 0.103]	0.000	Fixed
Nausea *	5	95	471	0.205[0.171, 0.245]	0.000	Fixed
Toothache	1	1	19	0.053[0.007, 0.294]	0.005	Fixed
Vomiting	4	52	370	0.154[0.093, 0.244]	0.000	Random
General disorders and administration site conditions						
Fatigue	5	92	418	0.222[0.134, 0.344]	0.000	Random
General physical health deterioration (general disorders and administration site conditions, other)	1	5	99	0.051[0.021, 0.116]	0.000	Fixed
Asthenia (fatigue) #	4	123	452	0.277[0.154, 0.447]	0.012	Random
Pyrexia (fever)	1	1	19	0.053[0.007, 0.294]	0.005	Fixed
Malaise	1	1	19	0.053[0.007, 0.294]	0.005	Fixed
Hepatobiliary disorders						
Hepatic function abnormal (hepatic failure) *	1	14	65	0.215[0.132, 0.332]	0.000	Fixed
Infections and infestations						
Paronychia	3	35	219	0.161[0.118, 0.216]	0.000	Fixed
Herpes zoster (shingles)	1	1	19	0.053[0.007, 0.294]	0.005	Fixed
Upper respiratory tract infection (upper respiratory infection)	1	3	19	0.158[0.052, 0.392]	0.008	Fixed
Urinary tract infection	3	38	265	0.144[0.107, 0.192]	0.000	Fixed
Urosepsis (infections and infestations, other)	1	3	99	0.030[0.010, 0.090]	0.000	Fixed
Injury, poisoning, and procedural complications						
Contusion (bruising)	1	1	19	0.053[0.007, 0.294]	0.005	Fixed
Investigations						
Alanine aminotransferase increased	2	20	120	0.167[0.110, 0.244]	0.000	Fixed
AST increased (aspartate aminotransferase increased)	1	3	19	0.158[0.052, 0.392]	0.008	Fixed
Increase in γ-glutamyltransferase (GGT increased)	1	3	99	0.030[0.010, 0.090]	0.000	Fixed
Weight decreased (weight loss)	1	17	101	0.168[0.107, 0.254]	0.000	Fixed
Metabolism and nutrition disorders						
Decreased appetite (anorexia) #	5	143	471	0.306[0.223, 0.404]	0.000	Random
Hyperphosphatemia #	6	331	483	0.682[0.594, 0.758]	0.000	Random
Hyponatremia	2	13	118	0.113[0.067, 0.186]	0.000	Fixed
Musculoskeletal and connective tissue disorders						
Arthralgia	1	7	65	0.108[0.052, 0.209]	0.000	Fixed
Back pain	1	8	65	0.123[0.063, 0.227]	0.000	Fixed
Muscle spasms (muscle cramp)	1	7	65	0.108[0.052, 0.209]	0.000	Fixed
Nervous system disorders						
Dysgeusia *	4	113	370	0.308[0.263, 0.357]	0.000	Fixed
Psychiatric disorders						
Insomnia	1	2	19	0.105[0.026, 0.337]	0.004	Fixed
Psychiatric disorders	1	2	19	0.105[0.026, 0.337]	0.004	Fixed
Renal and urinary disorders						
Acute kidney injury	1	6	99	0.061[0.027, 0.128]	0.000	Fixed
Hematuria	1	10	99	0.101[0.055, 0.178]	0.000	Fixed
Cystitis (Renal and urinary disorders, other)	1	1	19	0.053[0.007, 0.294]	0.005	Fixed
Respiratory, thoracic, and mediastinal disorders						
Rhinitis allergic (allergic rhinitis)	1	2	19	0.105[0.026, 0.337]	0.004	Fixed
Cough	1	2	19	0.105[0.026, 0.337]	0.004	Fixed
Dyspnea	4	57	284	0.155[0.055, 0.364]	0.003	Random
Oropharyngeal pain	1	1	19	0.053[0.007, 0.294]	0.005	Fixed
Laryngeal pain (respiratory, thoracic and mediastinal disorders, other)	1	1	19	0.053[0.007, 0.294]	0.005	Fixed
Skin and subcutaneous tissue disorders						
Alopecia	5	109	471	0.229[0.158, 0.321]	0.000	Fixed
Nail disorder (skin and subcutaneous tissue disorders, other)	2	10	111	0.093[0.051, 0.164]	0.000	Fixed
Nail dystrophy (skin and subcutaneous tissue disorders, other)	3	43	387	0.115[0.086, 0.151]	0.000	Fixed
Onycholysis (skin and subcutaneous tissue disorders, other)	3	55	387	0.146[0.114, 0.185]	0.000	Fixed
Onychalgia (skin and subcutaneous tissue disorders, other)	1	1	19	0.053[0.007, 0.294]	0.005	Fixed
Dry skin *	5	141	471	0.306[0.265, 0.349]	0.000	Fixed
Dermatitis (skin and subcutaneous tissue disorders, other)	1	1	19	0.053[0.007, 0.294]	0.005	Fixed
Nail discoloration	1	1	19	0.053[0.007, 0.294]	0.005	Fixed
Palmar-plantar erythrodysesthesia syndrome	2	27	166	0.156[0.085, 0.269]	0.000	Random
Hand-foot syndrome (palmar–plantar erythrodysesthesia syndrome)	2	43	286	0.160[0.071, 0.321]	0.000	Random
Pruritus	1	2	19	0.105[0.026, 0.337]	0.004	Fixed
Rash maculo-papular	1	2	19	0.105[0.026, 0.337]	0.004	Fixed

* The top 5 adverse events with highest occurrence rate analyzed by the fixed model.

# The top 5 adverse events with highest occurrence rate analyzed by the random model.

When fixed-effects models were applied to grade ≥ 3 AEs analysis, hyponatremia was found to be the most common AE (8.3%, 95% CI 5.6%–12.2%), while abnormal hepatic function (7.7%, 95% CI 3.2%–17.2%), anemia (7.0%, 95% CI 4.9%–9.8%), asthenia (6.6%, 95% CI 4.5%–9.5%), and nail dystrophy (6.0%, 95% CI 3.4%–10.3%) were other major AEs. Regarding random-effects models, stomatitis (9.7%, 95% CI 6.1%–15.1%) and general physical health deterioration (7.0%, 95% CI 2.8%–16.5%) commonly occurred in grade ≥3 AEs, and these consequences are listed in **Table 3** and **Supplementary Material 2**.

**Table 3:** The grade ≥3 adverse events classified by CAC Table 5.0 and the details.

Adverse events	No. of studies	No. of AE	No. of patients	Event rate with 95% CI	*p*-value	Model
Blood and lymphatic system disorders						
Anemia *	4	30	452	0.070[0.049, 0.098]	0.000	Fixed
Eye disorders						
Cataract	1	2	99	0.020[0.005, 0.077]	0.000	Fixed
Dry eye	2	2	200	0.010[0.003, 0.039]	0.000	Fixed
Keratitis	1	3	99	0.030[0.010, 0.090]	0.000	Fixed
Gastrointestinal disorders						
Abdominal pain	2	10	252	0.040[0.022, 0.072]	0.000	Fixed
Colitis	1	2	99	0.020[0.005, 0.077]	0.000	Fixed
Constipation	2	2	200	0.010[0.003, 0.039]	0.000	Fixed
Diarrhea	3	10	212	0.052[0.028, 0.095]	0.000	Fixed
Dry mouth	1	1	99	0.010[0.001, 0.067]	0.000	Fixed
Stomatitis (mucositis oral) #	3	36	387	0.097[0.061, 0.151]	0.000	Random
Aphthous ulcer (mucositis oral)	1	2	99	0.020[0.005, 0.077]	0.000	Fixed
Nausea	2	2	200	0.010[0.003, 0.039]	0.000	Fixed
Intestinal obstruction (small intestinal obstruction)	1	7	187	0.037[0.018, 0.076]	0.000	Fixed
Vomiting	1	2	99	0.020[0.005, 0.077]	0.000	Fixed
General disorders and administration site conditions						
Fatigue	3	6	212	0.041[0.010, 0.153]	0.000	Random
General physical health deterioration (general disorders and administration site conditions, other) #	3	32	351	0.070[0.028, 0.165]	0.000	Random
Asthenia (fatigue) *	3	25	387	0.066[0.045, 0.095]	0.000	Fixed
Hepatobiliary disorders						
Hepatic function abnormal (hepatic failure) *	1	5	65	0.077[0.032, 0.172]	0.000	Fixed
Infections and infestations						
Paronychia	2	6	200	0.030[0.014, 0.065]	0.000	Fixed
Urinary tract infection	3	11	265	0.045[0.025, 0.079]	0.000	Fixed
Urosepsis (infections and infestations, other)	1	3	99	0.030[0.010, 0.090]	0.000	Fixed
Investigations						
Alanine aminotransferase increased	2	4	200	0.020[0.008, 0.052]	0.000	Fixed
AST increased (aspartate aminotransferase increased)	1	10	187	0.053[0.029, 0.097]	0.000	Fixed
Increase in γ-glutamyltransferase (GGT increased)	1	2	99	0.020[0.005, 0.077]	0.000	Fixed
Weight decreased (weight loss)	1	1	101	0.010[0.001, 0.067]	0.000	Fixed
Metabolism and nutrition disorders						
Decreased appetite (anorexia)	2	2	166	0.012[0.003, 0.048]	0.000	Fixed
Hyperphosphatemia	4	6	399	0.020[0.009, 0.044]	0.000	Fixed
Hyponatremia *	2	23	286	0.083[0.056, 0.122]	0.000	Fixed
Nervous system disorders						
Dysgeusia	2	5	164	0.031[0.005, 0.158]	0.000	Random
Renal and urinary disorders						
Acute renal failure (acute kidney injury)	1	2	65	0.031[0.008, 0.115]	0.000	Fixed
Acute kidney injury	1	2	99	0.020[0.005, 0.077]	0.000	Fixed
Hematuria	1	2	99	0.020[0.005, 0.077]	0.000	Fixed
Respiratory, thoracic and mediastinal disorders						
Dyspnea	3	11	387	0.030[0.017, 0.053]	0.000	Fixed
Skin and subcutaneous tissue disorders						
Nail disorder (skin and subcutaneous tissue disorders, other)	2	4	111	0.039[0.015, 0.099]	0.000	Fixed
Nail dystrophy (skin and subcutaneous tissue disorders, other) *	2	12	200	0.060[0.034, 0.103]	0.000	Fixed
Onycholysis (skin and subcutaneous tissue disorders, other)	3	7	265	0.029[0.014, 0.059]	0.000	Fixed
Palmar–plantar erythrodysesthesia syndrome	2	8	166	0.048[0.024, 0.093]	0.000	Fixed
Hand-foot syndrome (palmar–plantar erythrodysesthesia syndrome)	1	5	99	0.051[0.021, 0.116]	0.000	Fixed

* The top 5 adverse events with highest occurrence rate analyzed by the fixed model.

# The top 2 adverse events with highest occurrence rate analyzed by the random model.

### 3.4 Efficacy of erdafitinib

**Figure 2:**
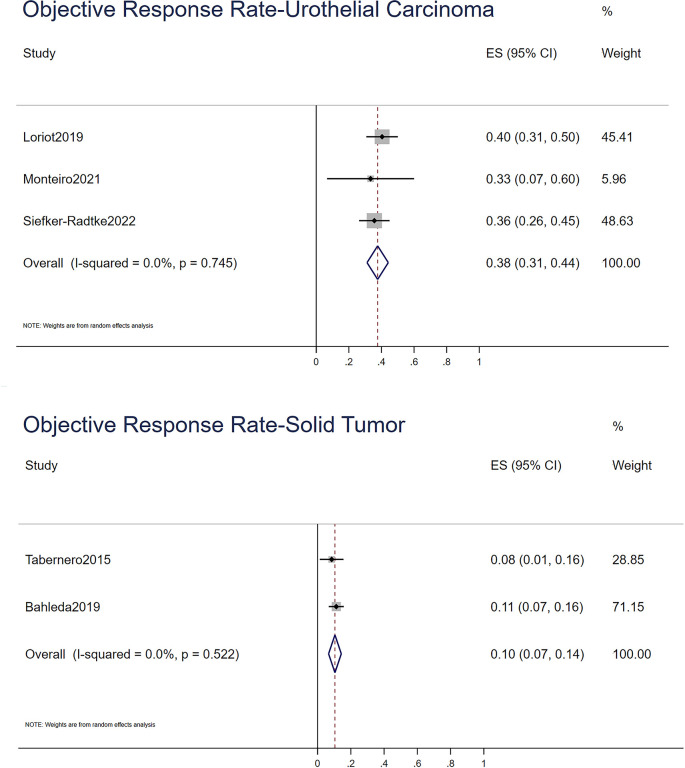
The objective response rate of urothelial carcinoma and solid tumor.

**Figure 3:**
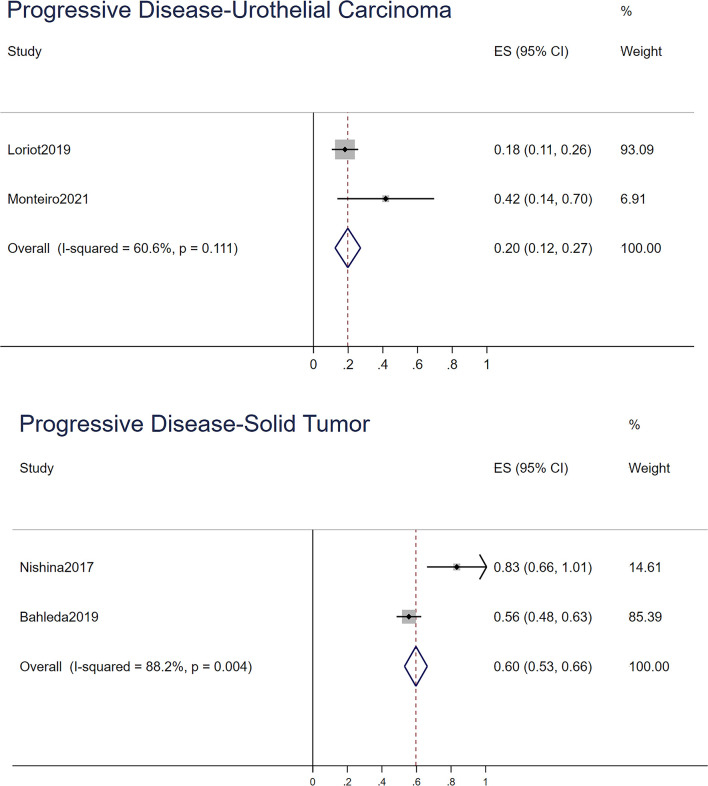
The progressive disease of urothelial carcinoma and solid tumor.

**Figure 4:**
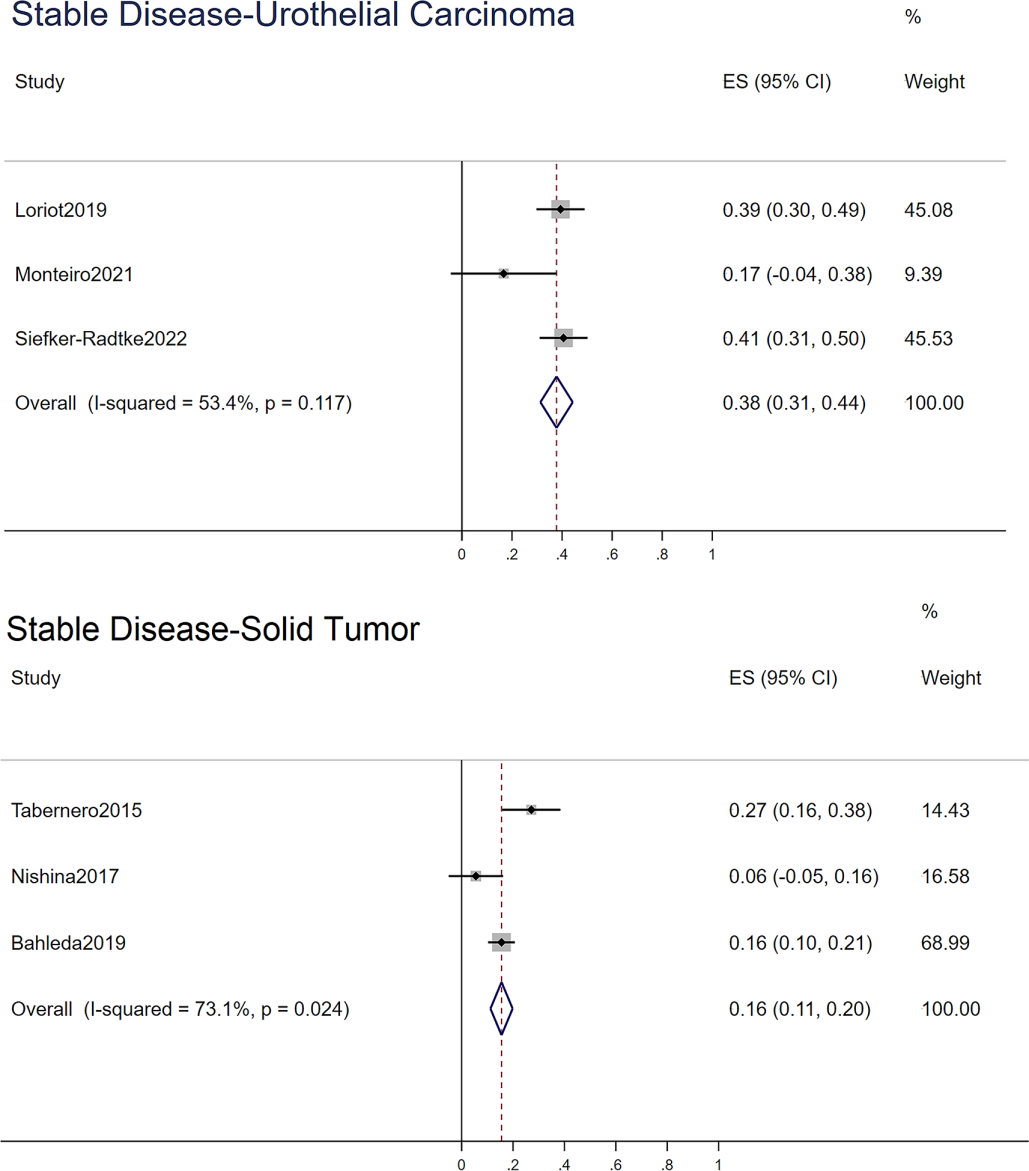
The stable disease of urothelial carcinoma and solid tumor.

For the ORR, stable disease rate, and progressive disease rate, STATA 15.0 was used to conduct a single-rate analysis. For solid tumors and urothelial carcinoma, we calculated their response rates separately, as shown in **Figures 2**–**4**. In the study of urothelial carcinoma, a random-effects model was used to analyze stable disease and progressive disease rates, which were 0.36 (0.26–0.46) and 0.26 (0.04–0.48), respectively. For urothelial carcinoma, the ORR was 0.38 (0.31–0.44) for the fixed model. Similarly, the ORR of solid tumors was 0.10 (0.07–0.14), while the overall stable disease and progressive disease rates of solid tumors were 0.16 (0.06–0.26) and 0.68 (0.41–0.95), respectively. Other details are shown in **Table 4**.

**Table 4:** Efficacy profile of erdafitinib.

STUDY	Complete response (CR)	Partial response (PR)	Stable disease (SD)	Objective response rate (ORR)	Progressive disease (PD)	Median duration of response	Median progression-free survival (PFS)
Josep Tabernero, 2015	0	5/59	16/59	5/59	NA	NA	NA
Tomohiro Nishina, 2017	0	0	1/18	0	15/18	NA	NA
Bahleda, 2019	0	21/187	29/187	21/187	104/187	9.0 months	2.3 months
Y. Loriot, 2019	3/99	37/99	39/99	40/99	18/99	5.6 months (95% CI = 4.2–7.2)	NA
Monteiro, 2021	0	4/12	2/12	4/12	5/12	NA	NA
Arlene O Siefker-Radtke, 2022	0	36/101	41/101	36/101	NA	6.0 months (95% CI = 4.2–7.5	5.5 months (95% CI = 4.2–6.0

Only three trials provided data on the median duration of response and median progression-free survival (PFS). The most prolonged duration occurred in studies published in 2022, which had a median duration of response of 6.0 months, while the median PFS was 5.5 months. A continuous dose of 8 mg or 8 to 9 mg was used for treatment.

In Siefker-Radtke’s trial, the median PFS of FGFR mutation was longer than that of FGFR fusion. For the patients who presented both FGFR mutation and fusion, the median PFS was 6.9 months, while mutation (−)/fusion (+) was 2.8 months, and mutation (+)/fusion (−) was 5.6 months.

### 3.5 Assessment for risk of bias and publication bias

RevMan 5.4 was used to assess the risk of bias. However, they were all single-arm studies. In Loriot’s study, except for performance bias, which was assessed as high risk because of open label, the other aspects were assessed as low risk. Five nonrandomized studies were evaluated as low to moderate risk in the ROBINS-I assessment. Overall, the quality of the studies was satisfactory. Because there were fewer than 10 included articles, the meta-regression and funnel plot were not made.

## 4 Discussion

Based on the present findings, we conducted a meta-analysis to summarize six published clinical trials (22–27), comprehensively investigating the safety and efficacy of erdafitinib. Our review analyzed the ORR, stable disease rate, and progressive disease rate of UC and other solid tumors separately. As mentioned in the *Characteristics of studies* section, some articles reported the outcomes regardless of UC and other solid tumors. Thus, we calculated the efficacy of UC and solid tumors separately by the trials that were specific to UC and the rest. Finally, we indicated that erdafitinib had a more satisfactory effect in UC than in solid tumors, with a higher ORR and lower progression rate.

Based on the development of next-generation DNA sequencing, it is now easy to determine the genetic alteration type of tumors. The effect of erdafitinib is surprising in some specific categories of FGFR gene alteration like *FGFR3-TACC3*. In Loriot’s trial, 4 of 11 patients responded to erdafitinib. All four patients had *FGFR3:TACC3v1* gene alteration (a specific kind of gene fusion). In Tabernero’s article, patients with *FGFR3-TACC3* tended to have greater response or tumor shrinkage than patients with other gene alterations. This phenomenon can be explained by a fusion of FGFR3 and TACC3, which contributed to constitutive tyrosine kinase activation and disruption of mitotic activity (28). Nevertheless, in addition to mutation and fusion, amplification may also occur. The trial (25) indicated that patients with FGFR mutations/fusion/co-alterations had significantly higher ORRs (12/27) than those with FGFR amplification (only 2/23 patients responded). Previous reviews have also found that for FGFR inhibitors, qualitative FGFR1–3 alterations such as mutation and rearrangement are more sensitive to drugs, and quantitative alterations like gene amplification rarely exhibit clinical activity (29). This might be because amplification leads to oncogene redundancy, which can lead to the overexpression of related proteins and initiate downstream signaling that promotes carcinoma proliferation and survival. Some studies have demonstrated that redundant oncogenes were associated with immune escape, which reduced or nullified the effect of FGFR SMi (30, 31).

In the study of AEs, we indicated that the most common all-grade AEs were hyperphosphatemia, stomatitis, dry mouth, dysgeusia, and diarrhea. Hyperphosphatemia occurred in more than half of the patients; however, all of them were grade 1–2. The most common grade ≥3 AEs were stomatitis, hyponatremia, and abnormal hepatic function. Generally, severe AEs were relatively rare.

For gastrointestinal AEs such as diarrhea, stomatitis, dry mouth, decreased appetite, nausea, vomiting, and abdominal pain, most can be controlled with symptomatic treatments (32). There have been no trials that reported drug withdrawal because of gastrointestinal AEs.

In the remaining AEs, hyperphosphatemia needed to be noted for its high occurrence and the possibility of causing reduction or withdrawal of erdafitinib. Hyperphosphatemia is the most common all-grade AE, occurring at 68.2%, related to fibroblast growth factor 23 (FGF-23) in bone metabolism (33, 34). FGF23 is a bone-derived mediator that maintains phosphate homeostasis, which inhibits the synthesis of 1,25-dihydroxyvitamin D3. Meanwhile, FGF-23 interacts with Klotho (which is the main structure of FGF receptor complex) to suppress renal phosphate reabsorption by decreasing the expression of the sodium-phosphate cotransporters NPT2A and NPT2C in the brush-border membrane of proximal tubule epithelial cells (35–37). FGFR1 co-expresses with Klotho, which increases the affinity of FGF23 for FGFR1 (38). A previous study suggested that Klotho was regulated by phosphaturia and that FGFR1 expression was modulated by FGF23. In a trial with a pan-FGFR inhibitor (PD173074), researchers found that the biologic activity of FGF-23 was counteracted, leading to hyperphosphatemia and high 1,25(OH)2 D3 (39). Therefore, the decrease in FGF-23 contributed to hyperphosphatemia and increased the production of calcitriol. In two phase I trials (NCT0103481 and NCT01962532), hyperphosphatemia appeared with 4 mg of erdafitinib. However, they both noted no dose-related changes in FGF23 values and vitamin D. Since no raw data on FGF23 content were provided, we did not perform a comprehensive analysis of FGF23. We suspected that the inconspicuous changes in FGF23 may be due to the following reasons. First, the sample size was small (the total number of patients was 82), and second, in NCT0103481, some patients reduced the dose of medication while others were given intermittent administration (27). These therapies may alleviate the inhibition of FGF23 by FGFR inhibitors. Therefore, FGFR23 did not show significant changes (40). Thus, the mechanism of erdafitinib-induced hyperphosphatemia needs further study. In patients with hyperphosphatemia, phosphate binders like sevelamer, acetazolamide, and sevelamer carbonate can be taken (25). Satisfactorily, the use of sevelamer has no significant effect on the pharmacokinetic parameters of erdafitinib.

In addition to hyperphosphatemia, central serous chorioretinopathy (CSC) was another AE mentioned in *FDA NEWS RELEASE: FDA approves first targeted therapy for metastatic bladder cancer*. In BLC2001, CSC occurred in 27 of 101 patients. A case report noticed that patients’ visual acuity changed from 20/25 OD and 20/15 OD to 20/20 OU after using erdafitinib (41). Meanwhile, in Tabernero’s study, one patient reported visual spots. As Jung et al. mentioned, this symptom might be caused by drug-induced pseudo-central serous chorioretinopathy (pCSC). However, it is worth noting though that primary CSC and paraneoplastic retinopathy (PNR, a retinopathy that occurs in patients with carcinomas) have the same symptoms (42). Therefore, a differential diagnosis is necessary for targeted treatment and appropriate prognosis prediction. For pCSC, retinopathy is often self-limited. The symptom disappears simultaneously or shortly after discontinuation of therapy, which is the most significant feature. The main difference of pathology between true CSC and PNR is that the former has typical features for lipofuscin irregularities, and the latter has progressive lesion (42). The included trials in this review showed that the dose of erdafitinib had no noticeable difference in the occurrence rate of retinopathy disease, with rates of 15/60 (25%) in the 8-mg QD group and 12/41 (29%) in the 9-mg QD group. After dose interruption, reduction, or shutoff, 17 of 27 patients were solved. After resolving detachment of retinal pigment epithelium (RPE), a grade 3 retinopathy patient recurred as grade 2. A similar phenomenon also occurred in Bahelda’s article, which indicated that after dose interruption, the pathological changes of the retina reversed except for the patients who had grade 1–2 retinopathy. However, grade 1–2 retinopathy events have not been solved in some patients. We have no accurate conclusions about why mild retinal damage still exists.

We suspected that this is related to other pathways downstream of FGFR, like MAPK. Some studies have indicated that MEK inhibitors have a toxic effect on RPE (43, 44), which leads to retinal-related AEs. Other studies have formulated some hypotheses. For instance, the Wnt/β-catenin signaling pathway can promote the proliferation of RPE and the accumulation of extracellular matrix. If the signaling pathway is impacted, RPE will become pathological (45). The pathological contraction and traction of the fibrocellular membranes cause retinal detachment (46). Another hypothesis states that FGFR-1 and FGFR-2 increase L-type Ca2+ channel activity in retinal pigment epithelial cells, and consequently promote the secretion of vascular endothelial growth factor A (VEGF-A), which plays a critical role in neovascularization. Decreasing visual acuity might cause that (47).

Nevertheless, this hypothesis for mild retinal damage requires further evidentiary support. Because pCSCs are self-limited, we do not suggest physicians use additional drugs other than closely observing and reducing the dose accordingly.

Presently, erdafitinib is being used with other drugs for clinical treatments, for example, combined with the PD-1 inhibitor cetrelimab (NCT03473743). More RCTs comparing erdafitinib to intravesical chemotherapy in non-muscle-invasive bladder cancer are ongoing. The unsatisfactory effect caused by FGFR gene amplification might be solved during new therapy, while AEs are alleviated by adjusting erdafitinib dosing.

There were some limitations in our article. For instance, the patients’ characteristics, the dose of erdafitinib, and FGFR gene alterations differ, which result in unavoidable heterogeneity. Moreover, restricted to the small number of included studies, we did not conduct meta-regression and funnel plots to assess the publication bias. Last but not least, all of the included articles are single-arm trials and lacked comparisons to other therapies.

As the first FDA-approved FGFR inhibitor to treat urothelial cancer, erdafitinib has a more satisfactory effect than traditional therapy. The most common AE is hyperphosphatemia, which occurs in grade 1–2 and can be controlled with sevelamer. Another AE worth discussing is pCSC. pCSC is caused by inhibiting the MAPK pathway, which needs to be distinguished from true CSC and PNR. Moreover, erdafitinib has rare severe AEs. In efficacy analysis, erdafitinib can increase the PFS significantly, among which, patients with FGFR mutations have a better response than those with fusions, while in FGFR gene fusion, FGFR3-TACC3 is the most sensitive gene alteration. Further studies on single-use and combined therapy of erdafitinib are ongoing, such as the phase III PROOF 302 trial (NCT04197986), which evaluates the efficacy of the FGFR1–3 inhibitor infigratinib in invasive urothelial carcinoma, which provides evidence for FGFR inhibitors in clinical decisions (48). After more clinical trials are published, the discoveries will be further improved.

## Data availability statement

The original contributions presented in the study are included in the article/**Supplementary Material.** Further inquiries can be directed to the corresponding authors.

## Author contributions

XYZ, HW, and JD wrote the original manuscript, and XLM designed and supervised this study. MHY made the major contribution to the data extracted. XHZ and FZ edited the language and improved figures and tables. All authors analyzed the data and edited the manuscript. All authors contributed to the article and approved the submitted version.
