# Supplementary material for: Safety and efficacy of the pan-FGFR inhibitor erdafitinib in advanced urothelial carcinoma and other solid tumors: A systematic review and meta-analysis
Source: Front Oncol. 2023 Jan 26;12:907377. doi: 10.3389/fonc.2022.907377 (PMC9909824; doi:10.3389/fonc.2022.907377)
Supplement: Supplementary file 1 [file Table_1.docx]

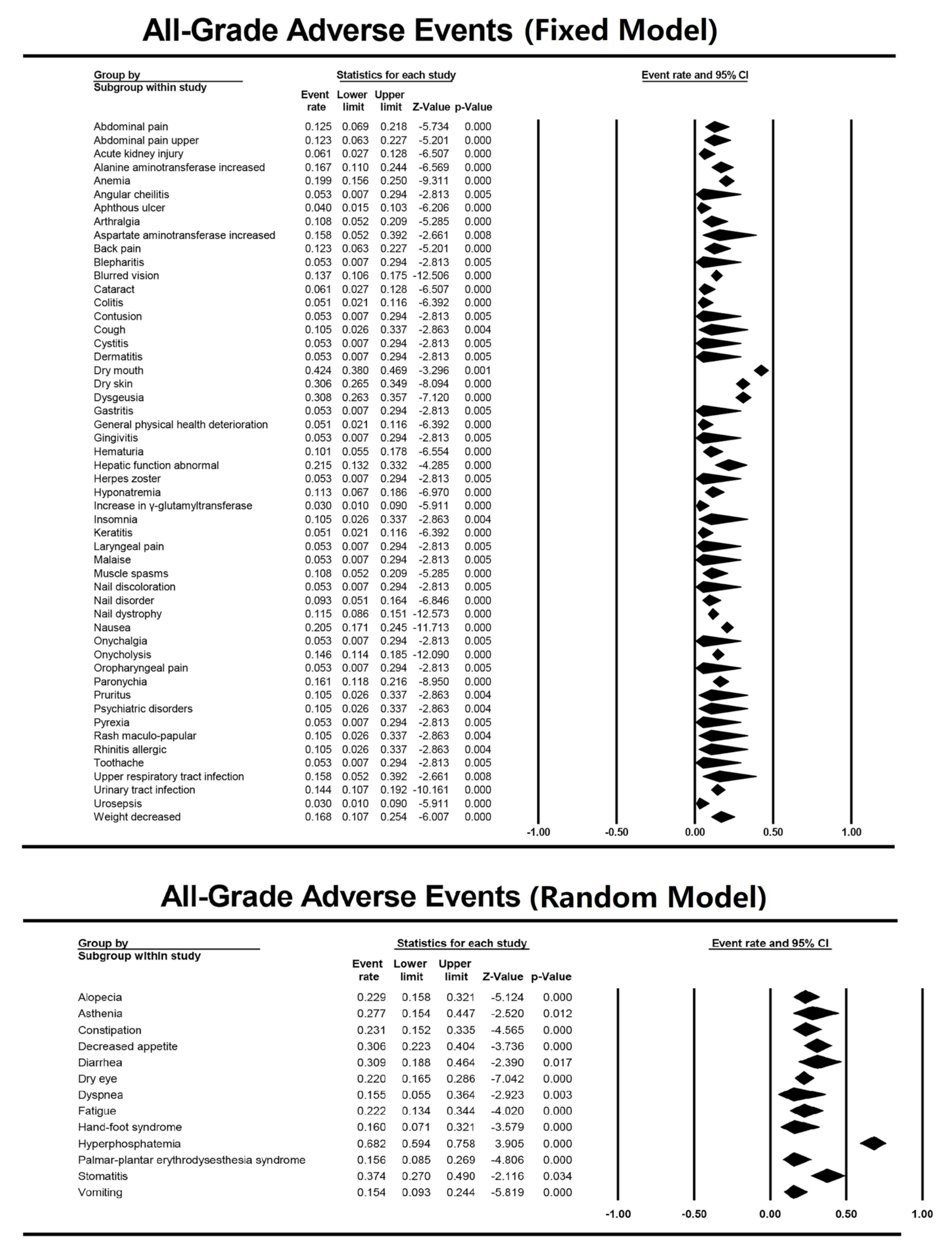


**Supplementary material 1.** The forest plots of all-grade adverse events in fixed and random models.
